# Menstrual changes following COVID-19 infection: A cross-sectional study from Jordan and Iraq

**DOI:** 10.1371/journal.pone.0270537

**Published:** 2022-06-29

**Authors:** Mohammad A. A. Al-Najjar, Ruaa R. Al-alwany, Firas M. Al-Rshoud, Rana K. Abu-Farha, Mohammed Zawiah

**Affiliations:** 1 Faculty of Pharmacy, Department of Pharmaceutics and Pharmaceutical Science, Applied Science Private University, Amman, Jordan; 2 Faculty of Medicine, Department of Obstetrics and Gynecology, The Hashemite University, Zarqa, Jordan; 3 Faculty of Pharmacy, Department Clinical Pharmacy, Applied Science Private University, Amman, Jordan; 4 Department of Pharmacy Practice, College of Clinical Pharmacy, University of Al-Hodeida, Al Hodeida, Yemen; 5 Discipline of Clinical Pharmacy, School of Pharmaceutical Sciences, Universiti Sans Malaysia, Penang, Malaysia; University of Hail, SAUDI ARABIA

## Abstract

**Purpose:**

COVID-19 infection is normally followed by several post-COVID effects. This study aimed to investigate to evaluate menstrual changes in females following COVID-19 infection, and to evaluate female perception about the effect of COVID-19 on their menstrual cycles.

**Methods:**

During this cross-sectional survey-based study, a convenience sample of 483 women from Jordan and from Iraq, who had infected with COVID-19 were invited to fill-out the study questionnaire.

**Results:**

The study was conducted on the females, with a median age 31 years old. Results showed that 47.2% of them (n = 228) suffered from a change in the number of days between two consecutive periods, as well as from a change in the amount of blood loss. Also, more than 50% of them believed that COVID-19 infection may cause changes in the amount of blood loss during the cycle (n = 375, 56.9%), and changes in the number of days between the two consecutive periods (n = 362, 54.2%).

Regression analysis showed that participants with higher educational level (bachelor or higher) (Beta = -0.114, P = 0.011), and those living in Iraq (Beta = -0.166, P<0.001) believed that COVID-19 has lower tendency to cause menstrual changes. In addition, non-married females (Beta = 0.109, P = 0.017), and those who are current smokers (Beta = 0.091, P = 0.048) believed that COVID-19 has higher tendency to cause menstrual changes.

**Conclusion:**

his study revealed that COVID-19 infection could affect the menstrual cycle for the females. Further prospective studies should be done to confirm these findings and evaluate how long these menstrual irregularities lasted.

## 1. Introduction

In December 2019, particularly in Wuhan China, several patients suffered from pneumonia of unknown etiology but their clinical implications were similar to SARS outbreak which spread in 2003. This new virus was named the severe acute respiratory syndrome coronavirus-2 (SARS-Cov-2), and currently the Coronavirus 19 (COVID-19). This disease has infected millions of victims worldwide [1]. For this reason, COVID-19 pandemic become the largest and the most diverse pandemic since the 1918 influenza pandemic, as the number of cases that are increasing on a daily base across 200 countries. The unprecedented spread of this pandemic disease is mainly due to its transformation through short distance human to human contact [2].

This infection can be asymptomatic or may range from mild to very severe symptoms, which might lead to death, especially for the elderly people as they are more susceptible to severe symptoms; while children tend to have mild symptoms [3–5]. COVID-19 symptoms usually start with fever, fatigue, dry cough, arthralgia, myalgia, sore throat, anosmia, diarrhea, vomiting and some neurological symptoms (such as headache, unstable walking, dizziness, and malaise) [4,6]. Some of these symptoms can last for weeks and months even after initial recovery and these symptoms, which are known as long term COVID-19 effects or post COVID-19 syndrome. Not only the hospitalized individuals may have these symptoms but also the individuals with mild infection. The five most common post COVID-19 effects are headache, fatigue, attention disorder, hair loss and dyspnea [7]. In addition, other studies indicated that one of the long term symptoms of COVID-19 is disturbances in the menstrual cycle of females [8–10].

Menstrual cycle is a biologically fundamental cycle for women controlled tightly by endocrine, autocrine and paracrine factors, with high variability in length (26–35 days) and variations in hormones levels during the cycle [11]. It is important for female reproductive state, and usually associated with three complaints including; pain, heaviness of bleeding and premenstrual syndrome (PMS) [11,12]. It highly affects the female psychology, mood and behaviors, but these effects have a broad range including; difference in pain sensitivity, emotional affect regulation, physical, cognitive and vocal disturbances [13].

Recently, researchers highlighted the relationship between COVID-19 infection and menstrual disturbances in females [8]. We hypothesize that most of the females have experienced changes in the menstrual cycle, and the highly educated females might consider these changes as post COVID-19 effects. In this study, we performed cross-sectional study aiming to evaluate menstrual changes in females following COVID-19 infection, and to evaluate female perception about the effect of COVID-19 on their menstrual cycles.

## 2. Method

### 2.1 Study design, settings, and participants

This is a cross-sectional survey-based study; that was conducted between 17^th^ January-1^st^ February 2022 in order to assess the menstrual changes in females following COVID-19 infection. During the study, a convenience sample of women were invited to participate on this study by sending the survey through social media (Facebook, and WhatsApp). The inclusion criteria were adult females who had infected with COVID-19. The survey required five minutes to be filled, and the participation was voluntary. Participants were asked to provide their electronic consent before filling the survey. If they want to participate on the survey, they will choose agree option. On the other hand, if they did not want to participate, they will choose disagree option.

### 2.2 Sample size calculation

The sample size was calculated based on the number of subjects per predictor needed to conduct linear regression analysis as recommended by Tabachnick and Fidell’s (5–20 subjects per predictor) [14]. Using 20 subjects per predictor, and since we have eight predictors, a minimum sample size of 160 was considered to be representative.

### 2.3 Survey instrument development and validation

An authenticated questionnaire that asses the effects of COVID-19 on the menstrual cycle were developed based on previous study [8]. The face and content validity were done on the draft questionnaire by a group of experts in the field. Then, the comments of experts were collected and revised. Laterally, the required modifications were made to the draft questionnaire.

The final questionnaire (**S1 Appendix**) consists of four sections: the first section assesses the demographic information of participants. The second section determines the medical information of participants. The third part assesses the menstrual cycle changes which occurred after COVID-19 infection (6 questions). And the last domain contains females’ perception toward the impact of COVID-19 infection on the menstrual cycle changes (7 statements). Perception section was evaluated using the following Likert scale (5: strongly agree, 4: agree, 3: neutral, 2: disagree, and 1: strongly disagree). For each patient, a perception score out of 5 was calculated for the seven perception statements. The survey was translated to Arabic and back translated to English, and it was distributed in the Arabic form, since Arabic is the mother tongue of the recruited women.

After that, the questionnaire was subjected to pilot testing on 10 women who had infected with COVID-19 to evaluate its structure, clarity, length, and give their overall impression, which resulted in several minor amendments to the original. The results of pilot testing were not included in the final analysis.

### 2.4 Ethical consideration

Ethics Committee provided Institutional Review Board (IRB) approval at The Hashemite University (Approval number:7/4/2021/2022). The World Medical Association Declaration of Helsinki guidance was followed in the study [15]. Participants were informed that their responses are anonymous, and their data will be kept confidential.

### 2.5 Statistical analysis

The final results were directly downloaded from the Google-Forms platform into the computer database as an Excel sheet. Then data file was converted and analyzed using IBM statistical package for social sciences (SPSS) (IBM SPSS Statistics, version 22.0, Chicago, Illinois). Descriptive analyses were presented as median ± interquartile range (IQR) for continuous variables, while frequency and proportions were used for categorical variables.

Simple linear regression was carried out to initially screen the independent variables that affect participants’ perception about the effects of COVID-19 on the menstrual cycle. Variables that were found to have P-value< 0.25 using univariate linear regression analysis, were entered into multiple linear regression analysis. Variables were selected after checking their independence, where tolerance values > 0.1 and Variance Inflation Factor (VIF) values were < 5 were selected to indicate the absence of multicollinearity between the independent variables in regression analysis. In the multiple linear regression analysis, variables that were independently affecting participants’ perception about the effects of COVID-19 on the menstrual cycle were identified. A P-value of ≤0.05 was considered statistically significant.

## 3. Results

During the study period, 483 female participants who were infected previously with COVID-19 were recruited, with a median age of 31 years (IQR = 10). The majority of the participants had a diploma or bachelor degree (n = 344, 71.3%), and around half of them were married (n = 248, 51.3%). Participants were recruited from both Jordan (n = 323, 66.9%) and Iraq (n = 160, 30.6%). Regarding the individual monthly income, around 54% of the females (n = 260, 53.8%) reported to have a monthly income less than 500$. Finally, 55.1% of the females (n = 266, 55.1%) reported to have a medical related degree. For more details about the socio-demographic of the study participants, refer to **Table 1**.

**Table 1:** Socio-demographic characteristics of the study sample (n = 483).

Parameter	Median (IQR)	n (%)
Age (years)	31.0 (10.0)	
Educational level ◦ School level or below ◦ Diploma ◦ Bachelor degree ◦ Graduate degree (Masters and PhD)		14 (2.9) 45 (9.3) 344 (71.2) 80 (16.6)
Marital status ◦ Married ◦ Non-married (single, widowed, divorced)		248 (51.3) 235 (48.7)
Current country of residence ◦ Jordan ◦ Iraq		323 (66.9) 160 (33.1)
Monthly income ◦ ≤500$/month ◦ 501–1000$/month ◦ 1001–2000$/month ◦ >2000 $/month		260 (53.8) 148 (30.6) 62 (12.8) 13 (2.7)
Do you have a medical degree? ◦ No ◦ Yes		217 (44.9) 266 (55.1)

IQR: Interquartile range.

Regarding participants’ medical information (**Table 2**), around half of the females (n = 236, 48.9%) have abnormal body weigh as following; 142 were overweight (29.4%), 93 were obese (19.3%), and one female was morbidly obese (0.2%). Moreover, around three-quarters of the females were non-smokers (n = 368, 76.2%), and the majority were vaccinated against COVID-19 (n = 438, 90.7%). Most of the participants (n = 346, 71.6%) were infected before receiving the vaccine while the remaining 137 females (28.4%) were infected after receiving the vaccine. Moreover, around one-third of the participants (n = 139, 35.0%) had received the seasonal influenza vaccine, and only 13.3% of them (n = 64) reported to have chronic diseases.

**Table 2:** Medical information of the study sample (n = 483).

Parameter	n (%)
BMI classes ◦ Under weight ◦ Normal weight ◦ Overweight ◦ Obese ◦ Morbidly obese ◦ Missing data	22 (4.6) 223 (46.2) 142 (29.4) 93 (19.3) 1 (0.2) 2 (0.4)
Smoking status: ◦ Current smoker ◦ Non-smoker ◦ Ex-smoker	98 (20.3) 368 (76.2) 17 (3.5)
Did you get COVID-19 vaccine? ◦ No ◦ Yes	45 (9.3) 438 (90.7)
You catch the COVID-19: ◦ Before getting the vaccine ◦ After getting the vaccine	346 (71.6) 137 (28.4)
Have you ever received influenza vaccine? ◦ No ◦ Yes	314 (65.0) 169 (35.0)
Do you have any chronic disease? ◦ No ◦ Yes	419 (86.7) 64 (13.3)

BMI: Body Mass Index.

The participated females were asked to describe the menstrual changes they experienced following their COVID-19 infection (**Table 3**), and results showed that 47.2% of them (n = 228) suffered from a change in the number of days between two consecutive periods whether an increase or decrease, as well as from a change in the amount of blood loss. In addition, 41.8% of the participants (n = 202) reported an increase or decrease in the length of menses. In addition, a lower percentage of females experienced a change in the pain which occurs just before or during menstruation (n = 203, 42.0%), missed period or suffered from period cessation after the infection (n = 109, 22.6%), and experienced bleeding between periods (n = 91, 18.8%).

**Table 3:** Menstrual cycle changes which occurred after COVID-19 infection (n = 483).

Parameter	n (%)
After infected with COVID-19, did you suffer from a change in the number of days between two consecutive periods ◦ No change ◦ Become closer and shorter ◦ Become longer	255 (52.8) 126 (26.1) 102 (21.1)
After contracting COVID-19, did you notice any change in the length of menses? ◦ No change ◦ Increase ◦ Decrease	281 (58.2) 97 (20.1) 105 (21.7)
After contracting COVID-19, did you notice any change in the amount of blood loss? ◦ No change ◦ Increase ◦ Decrease	253 (52.8) 134 (27.7) 96 (19.9)
After contracting COVID-19, have you experienced bleeding between periods (no matter how much)? ◦ No ◦ Yes	392 (81.2) 91 (18.8)
After contracting COVID-19, have you missed your period or suffered from cessation after the infection? ◦ No ◦ Yes	374 (77.4) 109 (22.6)
After contracting COVID-19, did you suffer from any change in pain which occurs just before or during menstruation? ◦ No ◦ Yes	280 (58.0) 203 (42.0)

**Fig 1:**
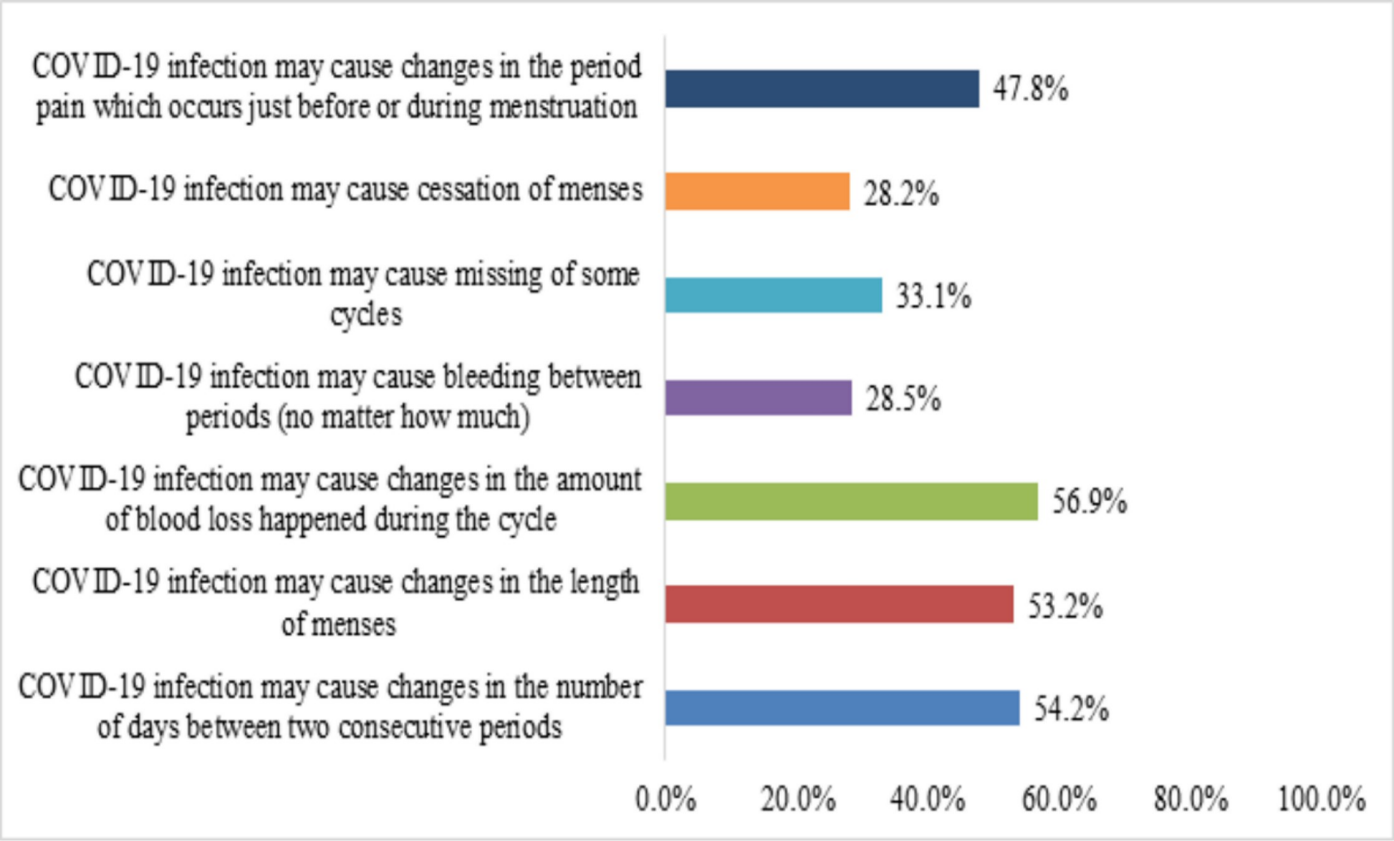
Participants’ perception towards the impact of COVID-19 infection on the menstrual change (n = 483).

Females were asked to express their perceptions towards the impact of COVID-19 infection on the menstrual change (**Fig 1**), and results showed that more than 50% of them believed that COVID-19 infection may cause changes in the amount of blood loss during the cycle (n = 375, 56.9%), changes in the number of days between the two consecutive periods (n = 362, 54.2%), and changes in the length of menses (n = 257, 53.2%). Also, 47.8% of the females (n = 231) believed COVID-19 infection may cause changes in the pain which occurs just before or during menstruation. Moreover, lower percentages of females agreed/strongly agreed that COVID-19 infection may cause missing of some cycles (n = 160, 33.1%), bleeding between periods (n = 138, 28.5%), cessation of menses (n = 136, 28.2%).

Lastly, the perception of the females towards the factors affecting the impact of COVID-19 infection on the menstrual change were investigated using univariate and multivariate linear regression analysis (**Table 4**). Results showed that participants with higher educational level (bachelor or higher) (Beta = -0.114, P = 0.011), and those living in Iraq (Beta = -0.166, P<0.001) have lower perception that COVID-19 causes menstrual changes. In addition, non-married females (Beta = 0.109, P = 0.017), and those who are current smokers (Beta = 0.091, P = 0.048) have higher perception that COVID-19 causes menstrual changes.

**Table 4:** Assessment of factors affecting participants’ perception towards the impact of COVID-19 infection on the menstrual change.

Parameter	Perception score			
	Beta	P-value#	Beta	P-value$
Age (years)	-0.017	0.709	----	----
Educational level ◦ Diploma or below ◦ Bachelor or Higher	Reference -0.113	0.013^	-0.114	0.011*
Marital status ◦ Married ◦ Non-married (single, widowed, divorced)	Reference 0.121	0.008^	0.109	0.017*
Current country of residence ◦ Jordan ◦ Iraq	Reference -0.201	<0.001^	-0.166	<0.001*
Monthly income ◦ ≤1000$/month ◦ >1000$/month	Reference 0.045	0.322	----	----
Do you have a medical degree? ◦ No ◦ Yes	Reference 0.063	0.168^	0.069	0.122
BMI classes ◦ Normal weight or below ◦ Overweight or above	Reference -0.015	0.747	----	----
Smoking status: ◦ Non-smoker/Ex-smoker ◦ Current smoker	Reference 0.150	0.001^	0.091	0.048*
Did you get COVID-19 vaccine? ◦ No ◦ Yes	Reference -0.018	0.696	----	----
Have you ever received influenza vaccine? ◦ No ◦ Yes	Reference 0.115	0.012^	0.066	0.139
Do you have any chronic disease? ◦ No ◦ Yes	Reference 0.010	0.834	----	----

^ Eligible for entry in multiple linear regression, # Using simple linear regression, $ Using multiple linear regression, * Significant at 0.05 significance level.

## 4. Discussion

During the previous two years the whole world faced a terrified waves of the COVID-19 pandemic that caused millions of life-losses and long-term effects. Post COVID-19 infection, the recovered people were worried about the loss of smell, loss of taste, loss of appetite, in addition to other symptoms. Recently, there have been scientific reports and even discussions on the social media that there is considerable number of the females infected with COVID-19 suffered from changes in the menstrual cycle [8–10]. The menstrual cycle generally is complex interactions of the hypothalamus, pituitary, ovaries, uterus, prostaglandins, and neuroendocrine factors. Therefore, the menstrual disturbances can be resulted from the disruption of any of these interactions [16].

Vitamins and other nutritional factors (such as vitamins C, D & B6) control these disturbances, and at the same time they have an important role in defending against microbial infectious included COVID-19 [16,17]. Vitamin D facilitates the production of antimicrobial peptides in the respiratory epithelium, reduces the possibility of viral infection and diminishes the severity of symptoms [18]. Because COVID-19 is a systemic inflammation, it will lower the circulation of 25 (OH)D resulting in vitamin D deficiency [17,19,20]. Low levels of vitamin D can cause irregular menstrual cycle including amenorrhea and oligomenorrhea [21,22], and this is mainly due to the direct effect of vitamin D on the circulating androgens [23]. Moreover, the low level of vitamin D can also worsen the premenstrual symptoms such as the pain, which will be stronger before or during menstrual cycle [24].

Furthermore, vitamin B6 plays a role on suppressing the severity of COVID-19 infection through activation of adaptive and innate immunity, reduces pro-inflammatory cytokines, enhance respiratory function, and prevent the hypercoagulability [25,26]. Therefore, the depletion of vitamin B6 during the COVID-19 infection in the females, increases level of estrogen in the blood and this lead to heavy and painful menstrual bleeding [16]. Vitamin C also plays an important role on the reproductive system as it is essential for the synthesis of collagen, steroids and peptides hormones, protection against oxidative damage [27]. Vitamin C can affect menstrual cycle indirectly, by playing a role on the absorption of other fat-soluble vitamins which control the cycle [12]. Moreover, it acts as promoting factor on the synthesis of estrogen and progesterone and improve hormonal levels, which lead to increase the thickness of endometrium, thus its deficiency may lead to heavy bleeding during the menstruation [28].

Almost half of the females participated, experienced changes in the menstrual cycle after COVID-19 infection, either in the number of the days between the consecutive cycles or amount of blood or the length of the menses (Table 3). Normally, these variations in the menstrual cycle can be affected by the psychological disturbances (particularly stress and depression) [29,30]. Such disturbances are well documented as long-term symptoms of COVID-19 infection [31]. In a previous study conducted in the United States, it was found that COVID-19-related stressors may also be a contributing factor in menstrual cycle changes. In that study, females showed a high perceived stress scale, at the same time they reported significant changes in their menstrual bleeding during the COVID-19 pandemic [32]. The majority of the participating females in our study, were vaccinated against COVID-19 (~91%), and 35% of them were also vaccinated against the seasonal flu (Table 3). Since, there is no evidence that the seasonal flu vaccination has an effect on the menstrual cycle [33], the females referred the changes that they experienced in the menstrual cycle to COVID-19 infection. Although it is well documented that females with higher body mass index experience more irregular menstrual cycle [34,35], the question in our survey was if they noticed changes compared to the period before COVID-19 infection. This suggests that the COVID-19 infection might have induced the effect of the high body mass index, or it might have the effect on the menstrual cycle solely, especially that almost half of the participating females had normal BMI or lower.

More than half of the females believe that COVID-19 infection may cause changes in the amount of blood loss during the cycle, changes in the number of days between the two consecutive periods, and changes in the length of menses (Table 4). Highly educated females believed that COVID-19 infection has lower tendency to cause disturbances in the menstrual cycle. This could be explained that highly educated have more updated knowledge about COVID-19 and its effects [36], and only few reports from the literature reported the association between COVID-19 infection and menstrual cycle disturbances [8,37,38]. Moreover, the non-married females and those who are currently smoking they believe that COVID-19 had higher tendency to cause changes on the menstrual cycle. Non-married females might be more aware on any menstrual cycle disturbances compared to married women for many reasons; such as the usage of contraceptive by married females [39]. The smoking females might have different reasons as they tend normally to accuse any health instabilities to any factor except the smoking habit.

The study has few limitations. First, the questionnaire was distributed through social media platforms, so, people without access to these applications would not be able to access this questionnaire. Second, this is a cross-sectional in nature and susceptible to recall bias; thus, further prospective longitudinal studies should be done to confirm these findings and evaluate how long these menstrual irregularities lasted. Third, in this study the psychological status of females during the COVID-19 pandemic was not assessed. Finally, we recruited a convenience sample of participants via social media, which may have introduced selection bias, limiting the generalizability of results to the general population.

## 5. Conclusion

This cross-sectional survey-based study revealed that COVID-19 infection could affect the menstrual cycle for the females within the age 21–42 years old. Further prospective studies should be done to confirm these findings and to evaluate how long these menstrual irregularities lasted.

## Supporting information

S1 Appendix (DOCX)
