# Supplementary material for: Menstrual changes following COVID-19 infection: A cross-sectional study from Jordan and Iraq
Source: PLoS One. 2022 Jun 29;17(6):e0270537. doi: 10.1371/journal.pone.0270537 (PMC9242447; doi:10.1371/journal.pone.0270537)
Supplement: S1 Appendix — (DOCX) [file pone.0270537.s001.docx]

**Menstrual Changes following COVID-19 Infection: A cross-sectional Study**

**Informed Consent Form**

**Dear participant**

Researchers from different countries are carrying out a research project with the purpose to assess the menstrual changes in females following COVID-19 infection.

To help protect your confidentiality, the surveys will not include information that will personally identify you, and will be used only for research purposes.

The procedure involves filling an online survey that will take approximately five minutes. Your participation is highly appreciated.

**ELECTRONIC CONSENT**: Please select your choice below.

If you are a female who were infected with COVID-19 for more than three months, you are invited to participate in our survey.

Clicking on the "agree" button below indicates that:

1. You have read the above information
2. You voluntarily agree to participate

If you do not wish to participate in the research study, please decline participation by clicking on the "disagree" button.

- Agree
- Disagree

**Menstrual Changes following COVID-19 Infection: A cross-sectional Study**

**Section 1: Demographic characteristics**

Age: ____________________ (years)

Current country of residence

- Jordan
- Other, specify ________________

Education level:

- School or below
- Diploma
- Bachelor
- Master/PhD

Marital status

- Married
- Single
- Others (widowed, divorced)

Personal income

- ≤500$/month
- 501-1000$/month
- 1001-2000$/month
- >2000 $/month

Do you have a medical-related degree?

- Yes
- No

Your body weight in kilograms __________________

Your height in cm _______________________

**Section 2. Medical information**

Smoking status:

- Smoker
- Non-smoker
- Ex-smoker

Did you get COVID-19 vaccine?

- No
- Yes

You catch the COVID-19:

- Before getting the vaccine
- After getting the vaccine

Have you ever received influenza vaccine?

- Yes
- No

Do you have any chronic disease?

- Yes
- No

**Section 3. Menstrual cycle changes which occurred after COVID-19 infection**

After infected with COVID-19, did you suffer from a change in the number of days between two consecutive periods

- No change
- Become closer and shorter
- Become longer

After contracting COVID-19, did you notice any change in the length of menses?

- No change
- Increase
- Decrease

After contracting COVID-19, did you notice any change in the amount of blood loss?

- No change
- Increase
- Decrease

After contracting COVID-19, have you experienced bleeding between periods (no matter how much)?

- No
- Yes

After contracting COVID-19, have you missed your period or suffered from cessation after the infection?

- No
- Yes

After contracting COVID-19, did you suffer from any change in pain which occurs just before or during menstruation?

- No
- Yes

**Section 4. Females perception towards the impact of COVID-19 infection on the menstrual changes**

| Statements | Strongly agree | Agree | Neutral | Disagree | Strongly disagree |
| --- | --- | --- | --- | --- | --- |
| COVID-19 infection may cause changes in the number of days between two consecutive periods |  |  |  |  |  |
| COVID-19 infection may cause changes in the length of menses |  |  |  |  |  |
| COVID-19 infection may cause changes in the amount of blood loss happened during the cycle |  |  |  |  |  |
| COVID-19 infection may cause bleeding between periods (no matter how much) |  |  |  |  |  |
| COVID-19 infection may cause missing of some cycles |  |  |  |  |  |
| COVID-19 infection may cause cessation of menses |  |  |  |  |  |
| COVID-19 infection may cause changes in the period pain which occurs just before or during menstruation |  |  |  |  |  |
